# A Deep Learning Model Integrating Ultrasound Images and Multidimensional Clinical Information for Differentiating Benign and Malignant Non-Mass Lesions

**DOI:** 10.3390/diagnostics16091380

**Published:** 2026-05-01

**Authors:** Weixian Huang, Zhiyu Xie, Zixuan Mo, Xing Tao, Yanhui Jiang, Dong Ni, Yanfeng Zhou, Jianxing Zhang

**Affiliations:** 1 The Second Clinical College, Guangzhou University of Chinese Medicine, Guangzhou 510000, China; 20231110667@stu.gzucm.edu.cn (W.H.); 20241110749@stu.gzucm.edu.cn (Z.M.); 2 School of Artificial Intelligence, Shenzhen University, Shenzhen 518060, China; 2510673011@mails.szu.edu.cn (Z.X.); taoxing2020@email.szu.edu.cn (X.T.); nidong@szu.edu.cn (D.N.); 3 National Engineering Laboratory for Big Data System Computing Technology, Shenzhen University, Shenzhen 518060, China; 4 Department of Ultrasound, Guangdong Provincial Hospital of Chinese Medicine, The Second Affiliated Hospital of Guangzhou University of Chinese Medicine, Guangzhou 510120, China; 20201110253@stu.gzucm.edu.cn

**Keywords:** ultrasound, breast non-mass lesions, artificial intelligence, deep learning, information fusion

## Abstract

**Background/Objectives:** This study aims to develop a deep learning (DL) model integrating ultrasound images and multidimensional clinical information to improve the diagnostic accuracy of breast non-mass lesions (NMLs). **Methods:** A total of 794 multicenter retrospective cases of NMLs were selected, stratified, and randomly divided into a training set (635 cases) and validation set (159 cases) at an 8:2 ratio. Multidimensional clinical information (including age, reproductive history, menstrual history, medical history, and findings from palpating the lesions) was incorporated to develop a DL model integrating ultrasound images and clinical data. To evaluate the diagnostic performance of the DL model, the area under the curve (AUC), accuracy, specificity, and sensitivity were employed. **Results:** The diagnostic model for NMLs integrating ultrasound images and multidimensional clinical information achieved an AUC of 0.8520 (95% CI: 0.7898–0.9068), F1 score of 0.7563, accuracy of 0.8176, sensitivity of 0.7031, and specificity of 0.8947. Its performance was superior to that of the model using only ultrasound images (AUC 0.8520 vs. 0.7571). SHAP analysis evaluating the reasons for the improved performance revealed that palpation with indistinct margins, abnormal axillary nodes, and older age were the three features with the highest contribution to predicting malignant risk. **Conclusions:** The DL model integrating ultrasound images and multidimensional clinical information demonstrated promising diagnostic performance in differentiating benign and malignant breast NMLs, suggesting the complementary value of multidimensional clinical information in the differential diagnosis of NMLs, though the reported AUC of 0.8520 is a preliminary internal estimate that awaits external validation.

## 1. Introduction

Breast cancer remains the most prevalent malignancy worldwide, with its incidence increasing annually, and it is a leading cause of cancer-related mortality [1,2]. Early detection and precise diagnosis are fundamental to effective clinical management, directly influencing treatment strategies and prognostic outcomes. Breast lesions are primarily classified on imaging as mass lesions or non-mass lesions (NMLs). According to the 2025 version of the American College of Radiology (ACR) Breast Imaging Reporting and Data System (BI-RADS), a non-mass lesion is a discrete finding that can be identified as distinctly different from normal tissue, is seen in three dimensions but lacks the discrete margination of a mass and cannot be assigned a specific shape. It is often subtle and may be detected only because the background tissue is disrupted [3]. Although NMLs account for only approximately 9.2% of all breast lesions, which is lower than that of mass lesions, their malignancy rate ranges from 10% to 54% [4,5,6]. The pathological spectrum includes benign conditions such as fibrocystic changes and sclerosing adenosis, as well as malignant entities like ductal carcinoma in situ and invasive lobular carcinoma [7,8,9]. Therefore, accurate differential diagnosis of benign and malignant NMLs is of significant practical importance for early breast cancer screening and subsequent patient management.

Ultrasound has become an indispensable modality for breast cancer screening, diagnosis, treatment guidance, and follow-up, owing to its advantages of low cost, lack of need for ionizing radiation, and capacity for real-time dynamic imaging [10,11]. In current clinical practice, the ultrasound diagnosis of NMLs primarily relies on physicians’ subjective interpretation of grayscale ultrasound features. Among these, linear or segmental distribution, associated microcalcifications, abnormal ductal changes, and posterior shadowing are considered suspicious findings suggestive of malignancy, whereas the presence of multiple small cysts is indicative of benign fibrocystic changes [12,13]. Previous studies have developed models integrating grayscale ultrasound features with multimodal ultrasound techniques to improve the diagnostic accuracy for NMLs [14,15,16,17]. However, due to the lack of a standardized definition for NMLs, the single-center design, and the relatively small sample sizes in these studies, their findings are limited. Some studies focused primarily on typical NMLs with calcifications [15] or predominantly intraductal lesions [14], resulting in study populations that lack representativeness. Consequently, the diagnostic performance of these models was suboptimal across different studies and populations. This issue becomes more pronounced when lesion characteristics are subtle or when benign and malignant features overlap, posing a key challenge to the accuracy of ultrasound in diagnosing NMLs. Therefore, it is necessary to adopt larger and more representative samples and novel techniques to enhance diagnostic accuracy.

The breakthrough progress of DL in image recognition has established it as a transformative tool for medical image analysis. In recent years, multiple studies have demonstrated the great potential of deep learning in the diagnosis of benign and malignant tumors. For example, Li et al. [18] proposed the DenseNet-II model by introducing the Inception structure into DenseNet, achieving a classification accuracy of 94.55% on mammography images. Al-Hejri et al. [19] adopted a hybrid architecture that integrates ensemble CNN features with a Vision Transformer encoder, reaching an accuracy of 98.58% in the binary benign-versus-malignant classification of mammography images. Subsequently, the same team extended a similar hybrid framework to cervical cytology image classification and also obtained excellent diagnostic performance [20]. Furthermore, Hussain and Toscano [21] achieved strong three-class classification performance on mammography images by optimizing multiple deep convolutional neural networks. However, most of the above studies are based on mammography, and AI models for breast lesions on ultrasound have also primarily focused on mass lesions [22,23], with limited exploration of DL-based diagnostic approaches specifically for NMLs. Li et al. [24] developed an NML detection and classification model using DL methods, which achieved promising performance in differentiating benign from malignant lesions, with an AUC of 0.837. While this study demonstrated the preliminary value of DL for NMLs, it relied primarily on training with mass lesions, only 228 NMLs were included, and relevant clinical features were not incorporated for multimodal joint learning, which limited its performance.

This study enrolled multicenter cases based on the standardized definition of NMLs in the 2025 ACR BI-RADS, with a large sample size and a comprehensive range of case types. It aimed to develop a DL model integrating ultrasound images with multidimensional clinical information for the automated classification of benign and malignant breast NMLs, and to evaluate the effectiveness and diagnostic performance of this model in improving the accuracy of differential diagnosis for NMLs, providing reliable references for clinicians in formulating precise treatment strategies and ultimately facilitating the individualized and precise clinical management of patients with breast NMLs. The developed model achieved high diagnostic performance, with an AUC of 0.8520, and demonstrated the complementary value of multidimensional clinical information, offering a promising tool for objective and standardized NML diagnosis.

## 2. Materials and Methods

### 2.1. Patients

The data of a total of 2738 consecutive patients who underwent breast ultrasound examination at the Dade Road General Hospital, University Town Hospital, and Fangcun Hospital of Guangdong Provincial Hospital of Traditional Chinese Medicine (National Regional Medical Center) from January 2015 to July 2025 were retrospectively collected. These cases were then confirmed as NMLs by two ultrasound physicians with over five years of experience; in the case of disagreement or uncertainty, a third physician with more than 10 years of experience in breast ultrasound diagnosis made the final decision. For each NML case, multidimensional clinical information, ultrasound images, and pathological results were collected. The multidimensional clinical information included age, age at menarche, menopausal status, age at menopause (for postmenopausal women), parity, age at first childbirth (for parous women), duration of breastfeeding (for parous women), lesion palpability, palpation margin and mobility for palpable lesions, personal history of breast cancer, abnormal axillary nodes, and nipple discharge.

**Figure 1:**
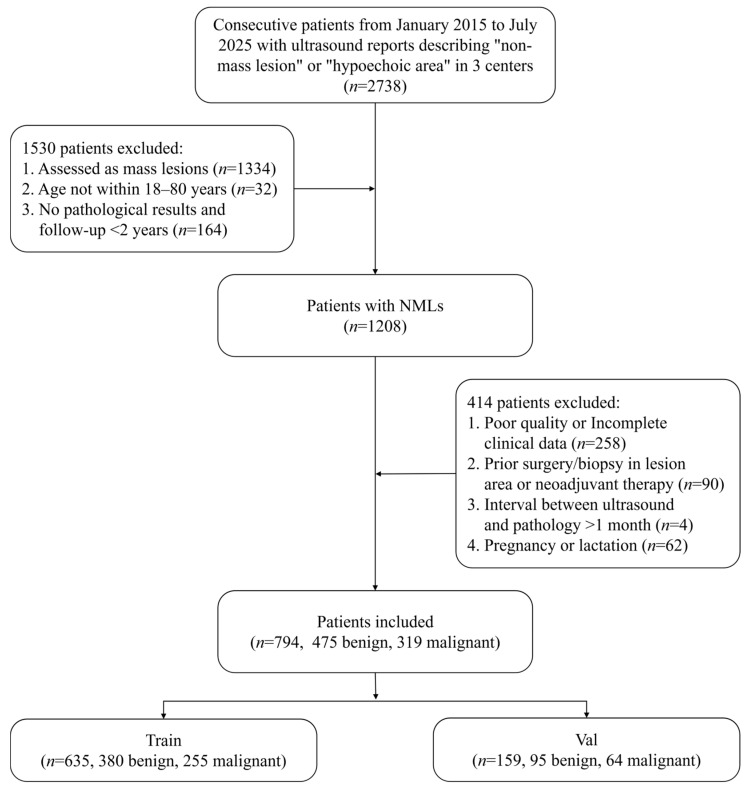
Flow chart of NML patient selection.

The inclusion criteria were (1) age between 18 and 80 years; (2) meeting the definition of NMLs with complete ultrasound images; and (3) having a definitive pathological diagnosis or imaging follow-up of at least 2 years showing no significant changes in lesion size and acoustic features. The exclusion criteria were (1) poor image quality or incomplete clinical data; (2) previous surgery or biopsy in the lesion area, or a history of neoadjuvant chemotherapy or endocrine therapy; (3) an interval between ultrasound examination and pathological confirmation exceeding one month; and (4) pregnancy, lactation, or severe cardiocerebral or psychiatric diseases. According to the inclusion and exclusion criteria, 794 NMLs from 794 patients were finally enrolled, with ages ranging from 21 to 80 years and a mean age of 46.20 ± 10.27 years. For each lesion, both radial and anti-radial ultrasound images were collected, resulting in a total of 1588 ultrasound images (Figure 1).

All cases were divided into benign and malignant groups based on pathological diagnosis or follow-up outcomes. They were then randomly divided into a training set (*n* = 635, including 380 benign and 255 malignant cases) and a validation set (*n* = 159, including 95 benign and 64 malignant cases) at an 8:2 ratio using stratified random sampling to ensure consistent proportions within both sets.

This study was approved by the Ethics Committee of Guangdong Provincial Hospital of Chinese Medicine, and the requirement for informed consent was waived (Approval No. ZE2023-427-01).

### 2.2. Equipment

Ultrasound images were acquired using the following equipment: GE LOGIQ–E9 (GE Healthcare, Wauwatosa, WI, USA, equipped with an ML6–15 linear array probe, center frequency 11 MHz); GE LOGIQ–E20 (GE Healthcare, Wauwatosa, WI, USA, equipped with an ML4–20 linear array probe, center frequency 11 MHz); and the Mindray A20 (Mindray, Shenzhen, China, equipped with an LM18–5WU linear array probe, center frequency 15.5 MHz). All ultrasound images were stored in Digital Imaging and Communications in Medicine (DICOM) format within the Picture Archiving and Communication System (PACS).

### 2.3. Gold Standard Determination

Pathological results were used as the diagnostic gold standard. For cases without pathological confirmation, a minimum of two years of imaging follow-up with no significant changes in lesion size and acoustic features was required to be considered benign [25].

Ultrasound images of all enrolled cases were independently annotated by two ultrasound physicians, each with over five years of experience in breast ultrasound and specialized training in reviewing 500 breast NML ultrasound images. The region of non-mass lesions (RONMLs) was segmented according to the following principles: (1) manually delineating along the identified lesion margins; (2) including all suspicious components within the delineated area; (3) simultaneously recording clinical information and benign/malignant labels during RONML annotation. In cases of disagreement or uncertainty, a third physician with more than 10 years of experience in breast ultrasound diagnosis made the final decision.

Axillary nodes were considered abnormal if any of the following criteria were met [26]: (1) greater than 10 mm in the maximum transverse dimension; (2) greater than 4 mm in the cortex thickness; (3) the presence of a lobulated cortical margin or absence of a hilum.

### 2.4. Inter-Rater Agreement Assessment

To ensure the consistency and reproducibility of the RONML annotation, an inter-rater agreement study was conducted on a randomly selected subset of 100 cases. The two annotators independently annotated the lesions following the same annotation principles described above. The Dice similarity coefficient (DSC) was calculated to quantify the overlap of the segmented regions, and a DSC ≥ 0.80 was considered to indicate good agreement.

### 2.5. Model Training

#### 2.5.1. Model Construction

**Figure 2:**
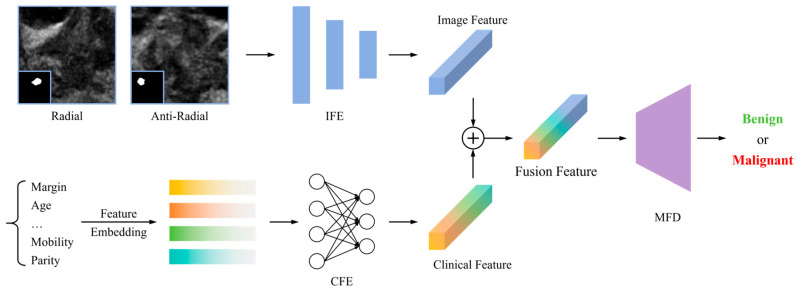
Model architecture diagram. ⊕ denotes channel concatenation of the two features.

This study constructed a case-level multimodal artificial neural network integrating ultrasound images and multidimensional clinical information for benign/malignant diagnosis of NMLs (Figure 2). The network consisted of three modules: an Image Feature Encoder (IFE), a Clinical Feature Encoder (CFE), and a Multi-modal Fusion Decoder (MFD). The IFE was implemented with an ImageNet-pretrained ResNet101. Each ROI-cropped ultrasound image was resized to 3 × 224 × 224 and passed through the standard ResNet101 stem (7 × 7 convolution with 64 filters, batch normalization, ReLU, and 3 × 3 max pooling), followed by four bottleneck residual stages containing 3, 4, 23, and 3 blocks with output channel dimensions of 256, 512, 1024, and 2048, respectively. The original fully connected classification layer of ResNet101 was removed and replaced with an identity mapping; after global average pooling, each image was represented by a 2048-dimensional feature vector. For each case, the feature vectors extracted from all available radial and anti-radial ultrasound images were averaged to obtain a case-level image representation F_img in R^2048^. The CFE received eight clinical variables, including age, menopausal status, reproductive history, history of breast cancer, abnormal axillary lymph nodes, lesion palpability, palpable margin, and lesion mobility. Each variable was first encoded into a 32-dimensional sinusoidal feature vector, and the eight encoded vectors were concatenated into a 256-dimensional clinical embedding. This embedding was then processed by two fully connected layers with 128 and 256 nodes, respectively, with ReLU activations, batch normalization after each layer, and a dropout of 0.3 after the first layer. The final clinical representation F_clin was therefore 256-dimensional. The MFD concatenated F_img and F_clin to form a 2304-dimensional fused feature vector. The fusion classifier consisted of two fully connected layers with 512 and 2 nodes, respectively; ReLU activation and a dropout of 0.5 were applied after the first fusion layer, and the final two outputs were converted to benign and malignant probabilities using softmax.

#### 2.5.2. Training Process

The model was implemented in PyTorch (version 2.1.1). The Adam optimizer and cross-entropy loss function were used for training, with a batch size of 16. The input images were resized to 224 × 224 and normalized using the ImageNet mean and standard deviation. In the sinusoidal positional encoding (SPE) block, each scalar clinical variable t_j was mapped to a 32-dimensional vector using sine and cosine functions at multiple frequencies: PE_{j,2k} = sin(t_j/10,000^{2k/d}) and PE_{j,2k + 1} = cos(t_j/10,000^{2k/d}), where d = 32 and k = 0, …, 15. The encoded vectors of the eight variables were concatenated to form a 256-dimensional clinical embedding before entering the two-layer CFE. In the random jitter cropping (RC) block, the nonzero pixels of the manually annotated lesion mask were first used to determine the lesion bounding box B = (x_min, y_min, x_max, y_max), with width w, height h, and center c. During training, a scale factor s was randomly sampled from U (1.2, 1.5), and the crop center was shifted by Delta x sampled from U (−0.2 w, 0.2 w) and Delta y sampled from U (−0.2 h, 0.2 h). The crop size was set to max (50, s × w) by max (50, s × h), clipped to the original image boundaries, and then resized to 224 × 224. One ROI crop was generated for each image. During validation and inference, deterministic cropping was used with s = 1.2 and no center jitter. If a valid mask was unavailable, the original image was resized directly. Additional image augmentations in training included random horizontal flipping and random rotation within −10 to 10 degrees.

### 2.6. Statistical Analysis

This study used SPSS 27.0 and R 4.4.1 software for statistical analysis. Continuous variables (including age, age at menarche, age at first childbirth, age at menopause, and breastfeeding duration) were tested for normality using the Shapiro–Wilk test. Variables with a normal distribution are expressed as means ± standard deviations, and comparisons between groups were conducted using the independent-samples *t*-test. Variables with a non-normal distribution are expressed as medians (P25, P75), and comparisons between groups were conducted using the Mann–Whitney U test. Categorical variables (including menopausal status, parity, abnormal axillary nodes, and nipple discharge, etc.) are described as frequencies (percentages), and comparisons between groups were conducted using the chi-square test. A *p*-value < 0.05 was considered statistically significant. Features with statistical significance were incorporated into the model together with ultrasound images for training. The diagnostic performance of the DL model was evaluated using the area under the curve (AUC), specificity, sensitivity, accuracy, and F1 score, with the optimal dynamic threshold selected based on the F1 score.

## 3. Results

### 3.1. General Patient Characteristics

A total of 794 patients with NMLs were included in this study. Of these, 723 (91.1%) were confirmed by pathological biopsy, and 71 (8.9%) were diagnosed based on follow-up of two years or longer. Among all cases, 319 (40.2%) were classified as malignant, and 475 cases (59.8%) as benign. The distribution types of all NMLs are detailed in Table 1. Focal distribution was the most common type, accounting for 56.1% of malignant cases and 84.8% of benign cases. Segmental and linear distributions were more frequently observed in malignant lesions (27.6% and 11.3%) compared to benign lesions (6.7% and 4.8%).

A comparison of clinical characteristics between patients with malignant and benign NMLs showed that age, menopausal status, parity, abnormal axillary lymph nodes, history of breast cancer, lesion palpability, palpation margin, and mobility were significantly different between groups (*p* < 0.05), as shown in Table 2. Specifically, the malignant group had a significantly higher median age [48 (41, 55) years] compared to the benign group [44 (38, 50) years]. The incidence of abnormal axillary nodes was markedly higher in the malignant group (33.9%) than in the benign group (1.5%). Additionally, the malignant group showed significantly higher proportions of lesions with indistinct palpation margins (88.1%) and fixed mobility (71.2%) compared to the benign group. No statistically significant differences were observed between groups regarding age at menarche, age at menopause, age at first childbirth, breastfeeding duration, or nipple discharge (*p* > 0.05), as presented in Table 3.

### 3.2. Inter-Rater Agreement

In the inter-rater agreement study, the mean Dice similarity coefficient was 0.886 (95% CI: 0.857–0.915; SD: 0.149), indicating good agreement between the two physicians.

### 3.3. Model Performance

**Figure 3:**
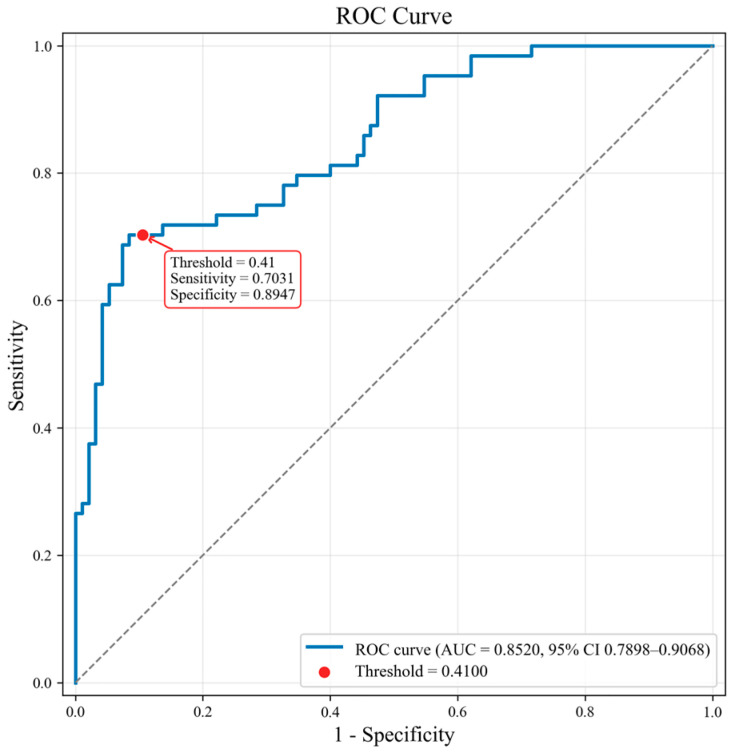
ROC curve of the proposed model. The diagonal dashed line represents the reference line of a random classifier (area under the curve = 0.5).

This study compared the performance improvements achieved by the baseline model using only ultrasound images versus the model with incremental incorporation of components (Table 4). In the validation set of 159 ultrasound examinations, the model using only ultrasound images achieved an area under the curve (AUC) of 0.7571, F1 score of 0.6767, accuracy of 0.7296, sensitivity of 0.6719, and specificity of 0.7474 for distinguishing benign from malignant NMLs. After incorporating multidimensional clinical information, the model’s performance improved, with an AUC of 0.8089 and F1 score of 0.7154. Following the application of sinusoidal positional encoding (SPE) and random jitter cropping centered on the lesion area (RC) to process and optimize the clinical information, the model achieved optimal performance, with an AUC of 0.8520. Finally, considering that our model is designed for clinical diagnostic assistance, where specificity is prioritized to reduce unnecessary biopsies, a dynamic threshold was applied to balance sensitivity and specificity, further improving the F1 score to 0.7563 (optimal diagnostic threshold: 0.4100), with an accuracy of 0.8176, sensitivity of 0.7031, and specificity of 0.8947 (Figure 3).

In order to evaluate the performance of various models, we also conducted a comparative experiment by using each backbone as the image feature encoder within our multi-modal fusion framework. Table 5 presents the performance of different models for the diagnosis of benign and malignant NMLs. ResNet101 achieved the highest overall performance, with an AUC of 0.8520 and an F1 score of 0.7563, and was therefore selected as the backbone for the final model.

### 3.4. SHAP Analysis

**Figure 4:**
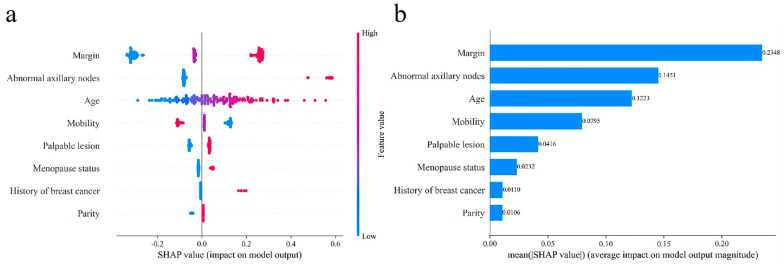
SHAP analysis of the eight clinical features incorporated in the model. (**a**) SHAP beeswarm plot, where each point represents the Shapley value for a feature and an individual sample, with the color indicating the feature value (red: high; blue: low). Positive SHAP values indicate a contribution toward malignancy prediction, while negative values indicate a contribution toward benign prediction. (**b**) SHAP bar plot.

The SHAP (Shapley Additive Explanations) method was applied to quantify the contribution and direction of each clinical feature in predicting benign and malignant NMLs. A positive SHAP value indicates that the feature drives the prediction toward malignancy, while a negative value suggests a tendency toward benignity. The results showed that all eight clinical features incorporated into the model influenced the predictions. Among them, an indistinct palpation margin and abnormal axillary nodes made the most substantial contributions to malignant prediction, followed by age, which emerged as an important clinical feature suggestive of malignancy in NMLs, as shown in Figure 4.

### 3.5. t-SNE Analysis

**Figure 5:**
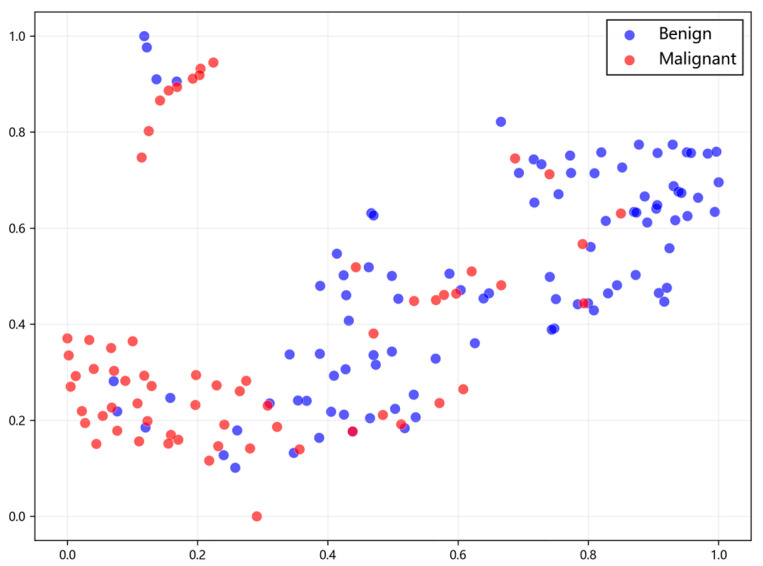
t-SNE visualization of fused feature representations.

The t-distributed stochastic neighbor embedding (t-SNE) method was used to embed the fused high-dimensional features into a two-dimensional space to visualize the distribution and clustering of different sample types in the low-dimensional space. This intuitively demonstrated the representational capability of the fused features extracted by the model in distinguishing benign from malignant NMLs, as shown in Figure 5. The majority of benign and malignant samples exhibited a relatively good clustering trend in the low-dimensional space, while a small proportion (approximately 18%) of benign and malignant samples overlapped, where discrimination was less clear.

### 3.6. Qualitative Results

**Figure 6:**
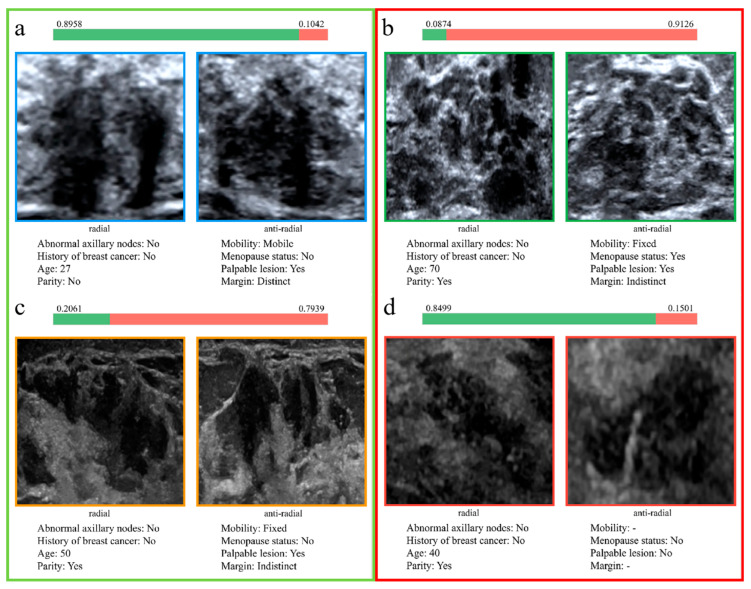
Examples of model performance on validation set cases. (**a**,**b**) Correctly classified cases. (**c**,**d**) Misclassified cases. Cases in green boxes (**a**,**c**) represent benign lesions, while cases in red boxes (**b**,**d**) represent malignant lesions. The green and red bars indicate the model’s predicted probabilities of benign and malignant for each case, respectively.

Correctly and incorrectly classified cases from the validation set were selected to analyze the reasons for accurate and erroneous classifications, as illustrated in Figure 6. Figure 6a,b show cases correctly classified, while Figure 6c,d present cases misclassified by the model.

## 4. Discussion

This study addressed the clinical challenge of overlapping benign and malignant features in NMLs and the difficulty of ultrasound diagnosis by constructing a deep learning model based on ResNet101. By innovatively integrating grayscale ultrasound images with multidimensional clinical information, the model achieved promising diagnostic performance in the validation set, with an AUC of 0.8520 and an F1 score of 0.7563. SHAP analysis further identified the palpation margin and abnormal axillary nodes as the core clinical features driving model predictions. This study is the first to demonstrate, using a large NML cohort (794 cases), that a deep learning model integrating multidimensional clinical information can significantly improve diagnostic accuracy, with decision-making logic closely aligning with clinical reasoning.

This study identified significant differences between malignant and benign NMLs in eight clinical characteristics, including age and menopausal status (Table 2). In contrast, previous studies with smaller sample sizes (<300 cases) [24,27,28] and predominantly screening-based cohorts [13] reported no such differences, likely due to their lower malignancy rates, which limited the ability to detect the variations. Given that this study population was derived from a clinical cohort with a malignancy rate of 40.2%, the larger sample size enabled the clearer identification of differences in clinical characteristics, and the study setting more closely reflects diagnostic practice.

Due to the lack of distinct morphology and clear margins in NMLs, the diagnostic performance of conventional BI-RADS classification using ultrasound is limited. In the study by Guo et al. [29], the AUC for differentiating benign from malignant NMLs using conventional ultrasound was 0.802. In comparison, our deep learning model achieved an AUC of 0.852 in the validation set. In a larger study by Park et al. [6], radiologists performed clinical BI-RADS assessment of NMLs using combined ultrasound and mammography, achieving AUCs ranging from 0.72 to 0.89 across different clinical indication subgroups (0.89 for the screening group, 0.88 for the diagnostic: work-up group, and 0.72 for the diagnostic: current breast cancer group). The AUC of this study’s model is slightly lower than that of radiologists in the screening and diagnostic: work-up groups, but higher than that in the diagnostic: current breast cancer subgroup. It should be noted that the study by Park et al. used combined information from ultrasound and mammography, whereas the present model relies solely on ultrasound features and clinical information. These results indicate that the model provides better discriminative ability for benign versus malignant NMLs than conventional ultrasound, approaching the performance of radiologists using combined mammography and ultrasound. Yang et al. [30] constructed a multimodal ultrasound model integrating SWE, CEUS, and SMI, achieving an AUC of 0.922; however, this approach requires multiple specialized ultrasound devices and complex operational procedures. Moreover, its small sample size and potential selection bias mean that its diagnostic efficacy, stability, and generalizability across larger populations and different types of NMLs remain to be further validated by prospective studies. Current AI diagnostic studies for NMLs have predominantly relied on imaging data alone. For instance, the MobileNet model developed by Li et al. [24] achieved an AUC of 0.837 in the NML validation set; however, this model was primarily trained on mass lesions, with only 228 NML samples, limiting its generalizability. In contrast, our model was developed using multicenter, large-sample cases collected based on the standardized definition of NMLs in the 2025 ACR BI-RADS, trained exclusively on NMLs, and integrated with relevant multidimensional clinical information, offering enhanced stability and generalizability. It should be noted that the sensitivity of the proposed model is only 0.7031, which implies that relying solely on the model for diagnosis may carry a certain risk of missed diagnoses. However, the model is intended for clinical diagnostic assistance, where specificity is prioritized to reduce unnecessary biopsy procedures. Furthermore, in a practical human–AI collaborative setting, the relatively low sensitivity can be partially compensated by the radiologist’s review. Notably, a relatively low sensitivity has also been reported in a previous deep learning study on NMLs. For instance, Li et al. [24] employed a MobileNet_448 model and achieved an AUC of 0.837, with a sensitivity of only 0.705 and a specificity of 0.803. This may be attributable to the imaging characteristics of NMLs, such as ill–defined margins and high heterogeneity.

This study innovatively integrated multidimensional clinical information to further enhance the diagnostic performance of the model (Table 4). After incorporating clinical information, the AUC improved by 12.5% compared to the model using only ultrasound images, fully demonstrating that clinical features can provide important complementary information to imaging characteristics, and their integration can effectively enhance the model’s ability to differentiate between benign and malignant NMLs. Shen et al. [31] preliminarily demonstrated the complementarity of clinical information in breast ultrasound AI research, and the study by Huang et al. [32] also showed that a dual-modality breast examination system integrating palpation information with ultrasound imaging can achieve higher diagnostic specificity. These findings provide an important reference for the present study, which incorporated multi-dimensional clinical information into the model and effectively improved its performance.

To further validate the robustness of our model architecture, we conducted a comparative experiment evaluating several mainstream backbone networks as image feature encoders within the multi-modal fusion framework (Table 5). The results demonstrated that deeper residual network (ResNet) architectures generally achieved better performance, with ResNet101 attaining the highest area under the receiver operating characteristic curve (AUC, 0.8520) and F1 score (0.7563). Specifically, compared with ResNet50, ResNet101 showed improvements in both accuracy (0.8176 vs. 0.8050) and specificity (0.8947 vs. 0.8842). This trend suggests that the enhanced representation capacity of deeper networks enables more effective extraction of the subtle and complex imaging patterns characteristic of NMLs. The superior performance of ResNet-based models in our study aligns with their well-established effectiveness across various medical image analysis tasks [33]. Notably, although Transformers have demonstrated potential to outperform CNNs on certain large-scale medical image datasets (e.g., CKHK-22) [34], their performance did not surpass that of ResNet101 in our preliminary analysis. This performance gap may be related to the relatively small size of the dataset. Previous studies have suggested that Transformers generally require large datasets to achieve their optimal performance [35,36]. In particular, the dataset used by Narasimha et al. [34], on which Transformers achieved excellent performance, contains more than 14,000 images, which is substantially larger than the NML dataset in the present study (1588 images). Furthermore, only one classic Transformer variant (ViT-B/16) was tested in this study. Therefore, future work should include more Transformer architectures and conduct further validation on larger-scale datasets.

The “black-box” nature of medical AI poses a significant barrier to its clinical translation. In this study, the SHAP analysis was applied for interpretability analysis of the model (Figure 4), identifying indistinct palpation margin, abnormal axillary nodes, and older age as the three core clinical features driving model decisions, with the direction of feature contributions closely aligning with clinical reasoning. An indistinct palpation margin contributed most substantially to malignant prediction, which is consistent with the histological characteristics of malignant NMLs, as they often exhibit infiltrative growth without a distinct capsule, resulting in indistinct margins on palpation. Abnormal axillary nodes directly indicate lesion invasiveness and serve as an important marker of metastatic spread. The median age was significantly higher in the malignant group than in the benign group, and the proportion of postmenopausal women was 1.7 times higher in the malignant group. These findings are highly consistent with the epidemiological characteristics of breast cancer, where incidence increases markedly with age, and cumulative estrogen exposure after menopause, along with aging breast tissue, is a major risk factor [1,37]. Furthermore, although hormone-related features such as menopausal status and parity showed smaller contributions, they remained important references for model prediction. This finding echoes the statistical analysis of clinical characteristics in this study and confirms that integrating multidimensional clinical information enables the model to form a more comprehensive risk assessment system. Compared with the AI-assisted reading tool developed by Lima et al. [38], our model not only achieves higher diagnostic performance but also offers clear interpretability, potentially assisting ultrasound physicians in more accurately identifying high-risk features of NMLs and improving the accuracy of diagnosis.

This study uses t-SNE dimensionality reduction visualization to intuitively demonstrate the representational capability of the fused features extracted by the model in distinguishing benign from malignant NMLs (Figure 5). The results showed a relatively favorable clustering trend between benign and malignant samples in the low-dimensional space, indicating that our model did not simply memorize the data but successfully learned discriminative diagnostic features capable of differentiating a considerable proportion of malignant NMLs from benign ones, thereby confirming the effectiveness of the model’s feature representation from the perspective of feature space. This finding is highly complementary to the results of the SHAP analysis: SHAP quantified the contribution of individual clinical features to the predictions, while t-SNE provided an overall visualization of how the fused features formed a discriminative spatial structure. However, the distribution of benign samples was relatively scattered and did not form clusters as compact as those of malignant samples. This may reflect the inherent diversity of their imaging features or suggest that there is room for improvement in the model’s ability to learn and condense benign patterns, pointing toward directions for optimizing feature representation in future research.

Additionally, this study presents representative cases from the validation set, including both correctly and incorrectly classified lesions (Figure 6). In Figure 6a, the lesion shows a focal distribution without malignant signs such as calcifications or posterior shadowing; the patient was young with distinct palpation margins and mobile mobility, and the model correctly diagnosed it as benign. In Figure 6b, the lesion demonstrates infiltrative growth involving the superficial fascia, with multiple microcalcifications within the lesion; the patient was older with indistinct palpation margins and fixed mobility, and the model correctly diagnosed it as malignant. In Figure 6c, the presence of a hyperechoic halo around the lesion and punctate calcifications within the duct represent suspicious malignant imaging features, combined with indistinct palpation margins, fixed mobility, and older age, leading the model to predict malignancy. In Figure 6d, although the lesion showed segmental distribution, there were no obvious malignant imaging features such as microcalcifications, and the lesion was non-palpable, which may have been the primary reason for the model’s misclassification as benign. This suggests that there is still room for optimization in the model’s decision boundary. Future efforts should focus on feature representation and fusion strategies for such “difficult cases” to further enhance diagnostic accuracy.

Although this study achieved promising results, several limitations should be acknowledged. First, despite incorporating interpretability analysis methods such as SHAP, the deep learning model remains a “black box” to some extent, and the specific features relied upon for benign–malignant differentiation cannot be fully elucidated. Second, the clinical features included in this study were limited to demographic and physical examination indicators; molecular biomarkers such as tumor markers and genetic testing were not integrated. Future research could explore the fusion of multi-omics information with imaging and clinical features to develop more precise diagnostic and prognostic assessment models for NMLs. Third, this study lacks external validation from independent institutions; consequently, the reported AUC of 0.8520 should be interpreted as a preliminary internal estimate that cannot yet be extrapolated to real-world clinical performance. The absence of a clinician-comparison benchmark further limits the assessment of added diagnostic value. Future multi-center prospective studies incorporating external validation cohorts and MRMC trials are required before any clinical deployment can be responsibly considered. Fourth, the source code is not publicly available due to a pending software copyright application. While detailed methodological descriptions are provided in Section 2.5, the absence of executable code restricts full independent replication.

## 5. Conclusions

In this study, a ResNet-based deep learning model integrating grayscale ultrasound images with multidimensional clinical information was constructed for differentiating benign from malignant breast NMLs. The model achieved promising diagnostic performance, with an AUC of 0.8520 in the validation set, along with high accuracy and specificity, effectively reducing the false-positive rate in NML diagnosis. SHAP analysis confirmed that an indistinct palpation margin, abnormal axillary nodes, and older age were the core clinical features indicative of malignancy, aligning with clinical reasoning, demonstrating good interpretability. These findings validate that multidimensional clinical information can effectively complement imaging characteristics, enhancing the diagnostic value of deep learning models and providing a novel technical approach for the objective and standardized diagnosis of NMLs.

## Figures and Tables

**Table 1:** The distribution of NMLs (*N* = 794).

Distribution	Malignant (*n* = 319)	Benign (*n* = 475)
Focal	179 (56.1)	403 (84.8)
Linear	36 (11.3)	23 (4.8)
Segmental	88 (27.6)	32 (6.7)
Regional	16 (5.0)	17 (3.6)

Note: Data are presented as frequency (percentage). Distribution types were defined according to the 2025 ACR BI-RADS lexicon.

**Table 2:** Statistically significant clinical characteristics of patients with NMLs (*N* = 794).

Characteristic	Malignant (*n* = 319)	Benign (*n* = 475)	*p* Value
Age (years), Median (Q1, Q3)	48 (41, 55)	44 (38, 50)	<0.001
Menopausal status, *n* (%)			<0.001
Yes	110 (34.5)	96 (20.2)	
No	209 (65.5)	379 (79.8)	
Parity, *n* (%)			0.003
Yes	292 (91.5)	401 (84.4)	
No	27 (8.5)	74 (15.6)	
History of breast cancer, *n* (%)			0.024
Yes	18 (5.7)	12 (2.5)	
No	301 (94.3)	463 (97.5)	
Abnormal axillary nodes, *n* (%)			<0.001
Yes	108 (33.9)	7 (1.5)	
No	211 (66.1)	468 (98.5)	
Palpable lesion, *n* (%)			<0.001
Yes	236 (74.0)	227 (47.8)	
Margin, *n* (%)			<0.001
Indistinct	208 (88.1)	119 (52.4)	
Distinct	28 (11.9)	108 (47.6)	
Mobility, *n* (%)			<0.001
Mobile	68 (28.8)	147 (64.8)	
Fixed	168 (71.2)	80 (35.2)	
No	83 (26.0)	248 (52.2)	

Note: Data are presented as median (Q1, Q3) for continuous variables or frequency (percentage) for categorical variables. Comparisons between malignant and benign groups were performed using the Mann–Whitney U test for continuous variables (age) and the chi-square test for categorical variables. Margin and Mobility refer to the palpable margin and mobility of NMLs and were analyzed only for the subset of palpable lesions. *p* < 0.05 for all comparisons, indicating statistically significant differences between the groups; therefore, these characteristics were included in the final deep learning model.

**Table 3:** Clinical characteristics without statistical significance in patients with NMLs (*N* = 794).

Characteristic	Malignant (*n* = 319)	Benign (*n* = 475)	*p* Value
Age at menarche (years) *	13 (12, 14)	13 (12, 14)	0.158
Age at menopause (years) *	50 (48, 52.25)	50 (46.25, 52)	0.336
Age at first birth (years) *	26 (24, 28)	26 (24, 28)	0.720
Breastfeeding (months) *	8 (4, 12)	8 (5, 12)	0.903
Nipple discharge, *n* (%)			0.846
Yes	24 (7.5)	34 (7.2)	
No	295 (92.5)	441 (92.8)	

Note: *: median (Q1, Q3). Data are presented as median (Q1, Q3) for continuous variables or frequency (percentage) for categorical variables. Comparisons between malignant and benign groups were performed using the Mann–Whitney U test for continuous variables and the chi-square test for categorical variables (nipple discharge). *p* > 0.05 for all comparisons, indicating no statistically significant differences between the groups; therefore, these characteristics were not included in the final deep learning model.

**Table 4:** Model performance at different optimization stages.

Clinical Information	SPE	RC	ACC	Sensitivity	Specificity	F1 Score	AUC
			0.7296	0.7031	0.7474	0.6767	0.7571
√			0.7610	0.6562	0.8316	0.6885	0.8057
√	√		0.7736	0.6875	0.8316	0.7097	0.8454
√	√	√	0.8176	0.7031	0.8947	0.7563	0.8520 *

Note: *: 95%CI = [0.7898, 0.9068]; clinical information refers to the factors that demonstrated statistically significant differences between benign and malignant NMLs in the univariate analysis (Table 2). SPE: sinusoidal positional encoding; RC: random jitter cropping. Checkmarks (✓) indicate which components were included in each model iteration. All models in this table use ResNet101 as the image feature encoder.

**Table 5:** Performance of various models.

Model	ACC	Sensitivity	Specificity	F1 Score	AUC
MobileNetV2	0.7484	0.6562	0.8105	0.6774	0.8192
DenseNet121	0.7610	0.6406	0.8421	0.6833	0.8094
ViT-B/16	0.7271	0.6094	0.8316	0.6555	0.8150
ResNet18	0.7421	0.6719	0.7895	0.6772	0.8174
ResNet34	0.7736	0.6406	0.8632	0.6949	0.8100
ResNet50	0.8050	0.6875	0.8842	0.7395	0.8388
ResNet101 (ours)	0.8176	0.7031	0.8947	0.7563	0.8520

Note: All models were trained and validated under identical conditions, incorporating the same clinical information and employing SPE and RC.

## Data Availability

The datasets used and/or analyzed during the current study are available from the corresponding author on reasonable request. The data are not publicly available due to privacy or ethical restrictions. The source code for the deep learning model is not publicly available due to a pending software copyright application. Detailed model descriptions are provided in Section 2.5. Code may be available upon reasonable request, subject to institutional intellectual property policies.

## Abbreviations

The following abbreviations are used in this manuscript:

NMLs	Non-Mass Lesions
AUC	Area Under the Curve
SHAP	Shapley Additive Explanations
DL	Deep Learning
BI-RADS	Breast Imaging Reporting and Data System
ACR	American College of Radiology
DICOM	Digital Imaging and Communications in Medicine
PACS	Picture Archiving and Communication System
IFE	Image Feature Encoder
CFE	Clinical Feature Encoder
MFD	Multi-modal Fusion Decoder
SPE	Sinusoidal Positional Encoding
RC	Random Jitter Cropping
t-SNE	t-Distributed Stochastic Neighbor Embedding
SWE	Shear Wave Elastography
SMI	Superb Microvascular Imaging
CEUS	Contrast-Enhanced Ultrasound
AI	Artificial Intelligence

## References

[1] Wilkinson L., Gathani T. (2022). Understanding breast cancer as a global health concern. Br. J. Radiol..

[2] Katsura C., Ogunmwonyi I., Kankam H.K., Saha S. (2022). Breast cancer: Presentation, investigation and management. Br. J. Hosp. Med..

[3] Newell M.S., Destounis S.V., Leung J.W.T., DeMartini W.B., Lee C.H., Eby P.R., Kasdan V.C., Attridge L.J., Pampillonia L.R., Chatfield M.B. (2025). ACR BI-RADS: Breast Imaging Reporting and Data System.

[4] Zhao Z., Hou S., Li S., Sheng D., Liu Q., Chang C., Chen J., Li J. (2022). Application of Deep Learning to Reduce the Rate of Malignancy Among BI-RADS 4A Breast Lesions Based on Ultrasonography. Ultrasound Med. Biol..

[5] Chang J.M., Leung J.W.T., Heacock L., Lee S.H., Moon W.K., Hooley R.J. (2026). Breast US: State of the Art. Radiology.

[6] Park V.Y., Choi J.S., Han K., Nahm S., Yoon J.H., Rho M., Yoon J., Kim M.J. (2025). Imaging Features and Diagnostic Performance of US in Nonmass Lesions with Varying Clinical Indications. Radiology.

[7] Choi J.S., Tsunoda H., Moon W.K. (2024). Nonmass Lesions on Breast US: An International Perspective on Clinical Use and Outcomes. J. Breast Imaging.

[8] Park K.W., Park S., Shon I., Kim M.J., Han B.K., Ko E.Y., Ko E.S., Shin J.H., Kwon M.R., Choi J.S. (2021). Non-mass lesions detected by breast US: Stratification of cancer risk for clinical management. Eur. Radiol..

[9] Choe J., Chikarmane S.A., Giess C.S. (2020). Nonmass Findings at Breast US: Definition, Classifications, and Differential Diagnosis. Radiographics.

[10] Mann R.M., Athanasiou A., Baltzer P.A.T., Camps-Herrero J., Clauser P., Fallenberg E.M., Forrai G., Fuchsjäger M.H., Helbich T.H., Killburn-Toppin F. (2022). Breast cancer screening in women with extremely dense breasts recommendations of the European Society of Breast Imaging (EUSOBI). Eur. Radiol..

[11] Guo R., Lu G., Qin B., Fei B. (2018). Ultrasound Imaging Technologies for Breast Cancer Detection and Management: A Review. Ultrasound Med. Biol..

[12] Tsunoda H., Moon W.K. (2024). Beyond BI-RADS: Nonmass Abnormalities on Breast Ultrasound. Korean J. Radiol..

[13] Ha S.M., Choi W.J., Han B.K., Kim H.H., Moon W.K., Kim M.J., Kim K., Yoen H., Kim H.J., Kim H. (2024). Assessment of Nonmass Lesions Detected with Screening Breast US Based on Mammographic Findings. Radiology.

[14] Kurt S.A., Taskin F., Kayadibi Y., Ozturk T., Adaletli I., Icten G.E. (2024). Role of Combining Grayscale Findings with Superb Microvascular Imaging and Shear Wave Elastography in Standardization and Management of NON-MASS Breast Lesions. Ultrasound Q..

[15] Kayadibi Y., Deger E., Kurt S.A., Ucar A.K., Adaletli I., Ozturk T., Kocael C.P., Velidedeoglu M., Icten G.E. (2023). The Diagnostic Role of Shear Wave Elastography and Superb Microvascular Imaging in the Evaluation of Suspicious Microcalcifications. J. Ultrasound Med..

[16] Sefidbakht S., Haseli S., Khalili N., Bazojoo V., Keshavarz P., Zeinali-Rafsanjani B. (2022). Can shear wave elastography be utilized as an additional tool for the assessment of non-mass breast lesions?. Ultrasound.

[17] Zhang F., Jin L., Li G., Jia C., Shi Q., Du L., Wu R. (2021). The role of contrast-enhanced ultrasound in the diagnosis of malignant non-mass breast lesions and exploration of diagnostic criteria. Br. J. Radiol..

[18] Li H., Zhuang S., Li D.-a., Zhao J., Ma Y. (2019). Benign and malignant classification of mammogram images based on deep learning. Biomed. Signal Process. Control.

[19] Al-Hejri A.M., Al-Tam R.M., Fazea M., Sable A.H., Lee S., Al-Antari M.A. (2022). ETECADx: Ensemble Self-Attention Transformer Encoder for Breast Cancer Diagnosis Using Full-Field Digital X-ray Breast Images. Diagnostics.

[20] Al-Hejri A.M., Al-Tam R.M., Sable A.H., Almuhaya B., Alshamrani S.S., Alshmrany K.M. (2025). A hybrid vision transformer with ensemble CNN framework for cervical cancer diagnosis. BMC Med. Inform. Decis. Mak..

[21] Hussain S.I., Toscano E. (2025). Optimized Deep Learning for Mammography: Augmentation and Tailored Architectures. Information.

[22] Gu Y., Xu W., Lin B., An X., Tian J., Ran H., Ren W., Chang C., Yuan J., Kang C. (2022). Deep learning based on ultrasound images assists breast lesion diagnosis in China: A multicenter diagnostic study. Insights Imaging.

[23] Sun Y., Qu Y., Wang D., Li Y., Ye L., Du J., Xu B., Li B., Li X., Zhang K. (2021). Deep learning model improves radiologists’ performance in detection and classification of breast lesions. Chin. J. Cancer Res..

[24] Li G., Tian H., Wu H., Huang Z., Yang K., Li J., Luo Y., Shi S., Cui C., Xu J. (2023). Artificial intelligence for non-mass breast lesions detection and classification on ultrasound images: A comparative study. BMC Med. Inform. Decis. Mak..

[25] Klein Wolterink F., Ab Mumin N., Appelman L., Derks-Rekers M., Imhof-Tas M., Lardenoije S., van der Leest M., Mann R.M. (2024). Diagnostic performance of 3D automated breast ultrasound (3D-ABUS) in a clinical screening setting-a retrospective study. Eur. Radiol..

[26] Ko K.H., Hsu H.H., Yu J.C., Peng Y.J., Tung H.J., Chu C.M., Chang T.H., Chang W.C., Wu Y.C., Lin Y.P. (2015). Non-mass-like breast lesions at ultrasonography: Feature analysis and BI-RADS assessment. Eur. J. Radiol..

[27] Lin M., Wu S. (2022). Ultrasound classification of non-mass breast lesions following BI-RADS presents high positive predictive value. PLoS ONE.

[28] Zhang W., Xiao X., Xu X., Liang M., Wu H., Ruan J., Luo B. (2018). Non-Mass Breast Lesions on Ultrasound: Feature Exploration and Multimode Ultrasonic Diagnosis. Ultrasound Med. Biol..

[29] Guo W., Wang T., Li F., Jia C., Zheng S., Zhang X., Bai M. (2022). Non-mass Breast Lesions: Could Multimodal Ultrasound Imaging Be Helpful for Their Diagnosis?. Diagnostics.

[30] Yang Z., Lyu Y., Yang Y., Xu C., Zhang K., Yang X., Chen F., Cheng L., Ren M. (2025). Application of Multimodal Ultrasound Combined With BI-RADS in Classification and Diagnosis of Non-Mass Breast Lesions. J. Clin. Ultrasound.

[31] Shen Y., Shamout F.E., Oliver J.R., Witowski J., Kannan K., Park J., Wu N., Huddleston C., Wolfson S., Millet A. (2021). Artificial intelligence system reduces false-positive findings in the interpretation of breast ultrasound exams. Nat. Commun..

[32] Huang X., Zhao S., Chen W., Sun B., Lin Z., Yang H. (2024). Evaluating the features of breast lesions identified by bimodal breast examination: A real-world study. Front. Oncol..

[33] Zhang Q. (2022). A novel ResNet101 model based on dense dilated convolution for image classification. SN Appl. Sci..

[34] Narasimha Raju A.S., Venkatesh K., Rajababu M., Kumar Gatla R., Jakeer Hussain S., Satya Mohan Chowdary G., Ganga Bhavani T., Kareemullah M., Algburi S., Majdi A. (2025). Colorectal cancer unmasked: A synergistic AI framework for Hyper-granular image dissection, precision segmentation, and automated diagnosis. BMC Med. Imaging.

[35] Lu Z., Liu C., Chang X., Zhang Y., Xie H. (2025). DHVT: Dynamic Hybrid Vision Transformer for Small Dataset Recognition. IEEE Trans. Pattern Anal. Mach. Intell..

[36] Willemink M.J., Roth H.R., Sandfort V. (2022). Toward Foundational Deep Learning Models for Medical Imaging in the New Era of Transformer Networks. Radiol. Artif. Intell..

[37] Konat-Bąska K., Matkowski R., Błaszczyk J., Błaszczyk D., Staszek-Szewczyk U., Piłat-Norkowska N., Maciejczyk A. (2020). Does Breast Cancer Increasingly Affect Younger Women?. Int. J. Environ. Res. Public Health.

[38] Lima I.R.M., Cruz R.M., de Lima Rodrigues C.L., Lago B.M., da Cunha R.F., Damiao S.Q., Wanderley M.C., Bitencourt A.G.V. (2025). Performance of AI-Based software in predicting malignancy risk in breast lesions identified on targeted ultrasound. Eur. J. Radiol..

